# Supplementary material for: Hybrid Mesoporous Silica Nanoparticles Grafted with 2-(tert-butylamino)ethyl Methacrylate-b-poly(ethylene Glycol) Methyl Ether Methacrylate Diblock Brushes as Drug Nanocarrier
Source: Molecules. 2020 Jan 3;25(1):195. doi: 10.3390/molecules25010195 (PMC6983194; doi:10.3390/molecules25010195)
Supplement: Supplementary file 1 [file molecules-25-00195-s001.pdf]

Hybrid Mesoporous Silica Nanoparticles Grafted with 2-(tert-butylamino)ethyl methacrylate-b-poly(ethylene glycol) methyl ether methacrylate Diblock Brushes as Drug Nanocarrier

Abdullah M Alswieleh[1],†,*, Abeer M Beagan[1],†, Bayan M Alsheheri[1], Khalid M Alotaibi[1], Mansour D Alharthi[1] and Mohammed S Almeataq[2]*

[1] Department of Chemistry, College of Science, King Saud University, Riyadh, Kingdom of Saudi Arabia.
[2] King Abdulaziz City for Science and Techenology, Riyadh, Kingdom of Saudi Arabia.
* Correspondence: A. Alswieleh, Email: aswieleh@ksu.edu.sa, M. Almeataq, Email: mmeataq@kacst.edu.sa
† These authors contributed equally.

Received: date; Accepted: date; Published: date

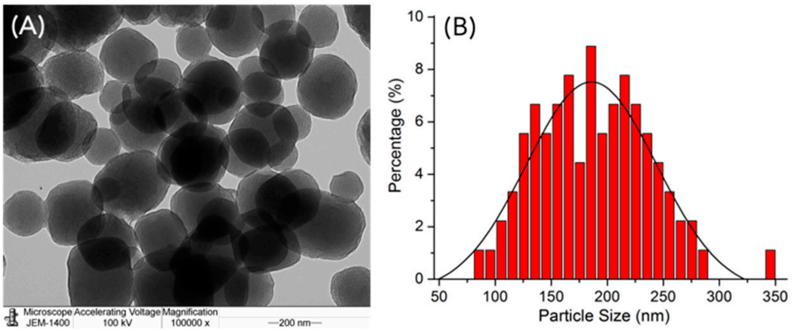

Figure S1: (A) TEM image of unmodified mesoporous silica nanoparticles. (B) The particle-size distribution of the fabricated nanoparticles.

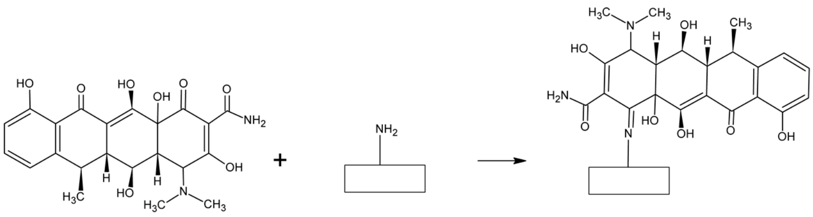

Scheme S1: Illustration the possible interaction between Doxy and amino groups in the MSNs surface.
